# Supplementary material for: Gossypitrin, A Naturally Occurring Flavonoid, Attenuates Iron-Induced Neuronal and Mitochondrial Damage
Source: Molecules. 2021 Jun 2;26(11):3364. doi: 10.3390/molecules26113364 (PMC8199700; doi:10.3390/molecules26113364)
Supplement: Supplementary file 1 [file molecules-26-03364-s001.zip › molecules-1212346-supplementary.pdf]

## Supporting Information

# Gossypitrin, A Naturally Occurring Flavonoid, Attenuates Iron-Induced Neuronal and Mitochondrial Damage

**María Ángeles Bécquer-Viart [1], Adonis Armentero-López [1], Daniel Alvarez-Almiñaque [1], Roberto Fernández-Acosta [2], Yasser Matos-Peralta [3], Richard F. D'Vries [4], Javier Marín-Prida [1] and Gilberto L. Pardo-Andreu [1,*]**

[1] Center for Research and Biological Evaluations, Institute of Pharmaceutical and Food Sciences, University of Havana, 222 St. #2317 b/ 23 and 31, La Coronela, La Lisa, CP 13600, La Habana, Cuba; mabecquer@ifal.uh.cu (M.A.B.-V.); adonis.armenteros@ifal.uh.cu (A.A.-L.); dani@ifal.uh.cu (D.A.-A.); javier.marin@ifal.uh.cu (J.M.-P.)

[2] Pharmacy Department, Institute of Pharmaceutical and Food Sciences, University of Havana, 222 St. #2317 b/ 23 and 31, La Coronela, La Lisa, CP 13600, La Habana, Cuba; roberto.fernandezac91@gmail.com

[3] Bioinorganic Laboratory, Faculty of Chemistry, University of Havana, Zapata and G, Vedado, CP 10400, La Habana, Cuba; yasser.matos@fq.uh.cu

[4] Facultad de Ciencias Básicas, Universidad Santiago de Cali, Calle 5 # 62-00, CP 76001, Cali, Valle del Cauca, Colombia; richard.dvries00@usc.edu.co

* Correspondence: gpardo@ifal.uh.cu; Tel.: +53-7271-8534

The $^{1}$H-NMR, $^{13}$C-NMR, and HSQC spectra of gossypitrin are shown in Figures S1, S2, and S3, respectively.

$^{1}$H NMR (400 MHz, DMSO-$d_6$) δ 8.07 (d, $J$ = 2.1 Hz, 1H), 7.91 (dd, $J$ = 8.6, 2.1 Hz, 1H), 6.99 (d, $J$ = 8.6 Hz, 1H), 6.26 (s, 1H), 4.83 (d, $J$ = 7.3 Hz, 1H), 3.75 (dd, $J$ = 9.6 Hz, 1H), 3.59 (dd, $J$ = 12.4 Hz, 1H), 3.46 – 3.25 (m, 38H).

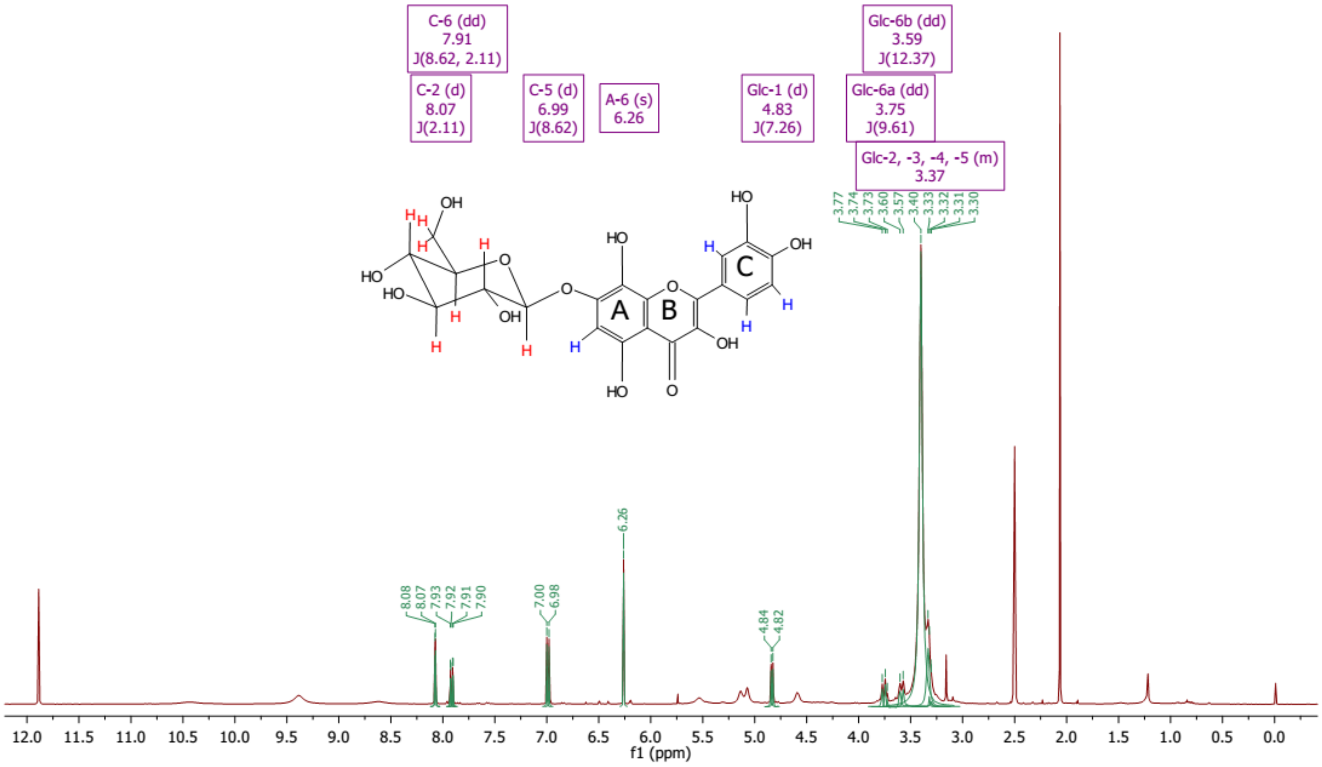


**Figure S1.** $^{1}$H-NMR (400 MHz) spectrum of gossypitrin in DMSO-$d_6$.

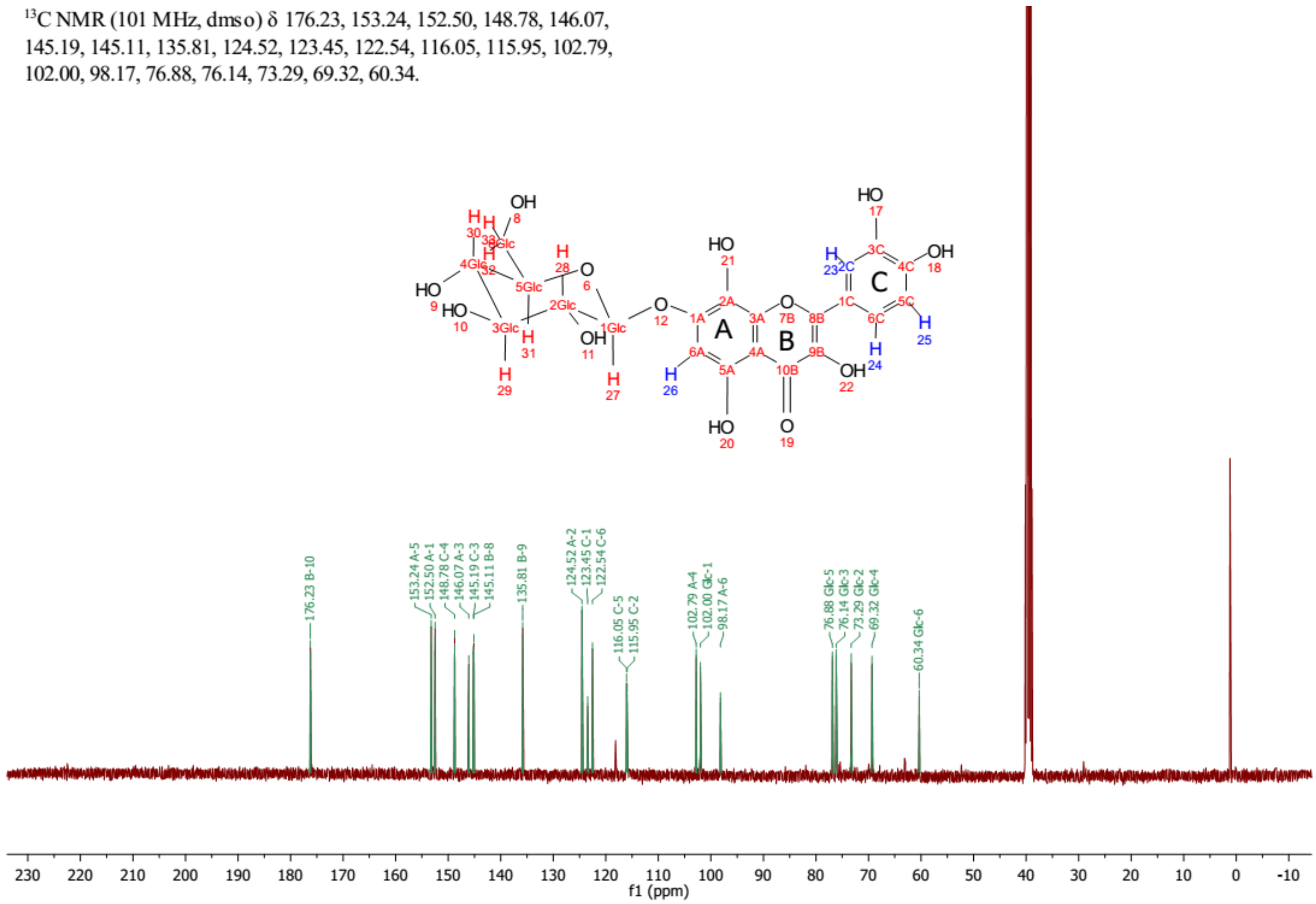


**Figure S2.** $^{13}C$-NMR (101 MHz) spectrum of gossypitrin in DMSO-$d_6$.

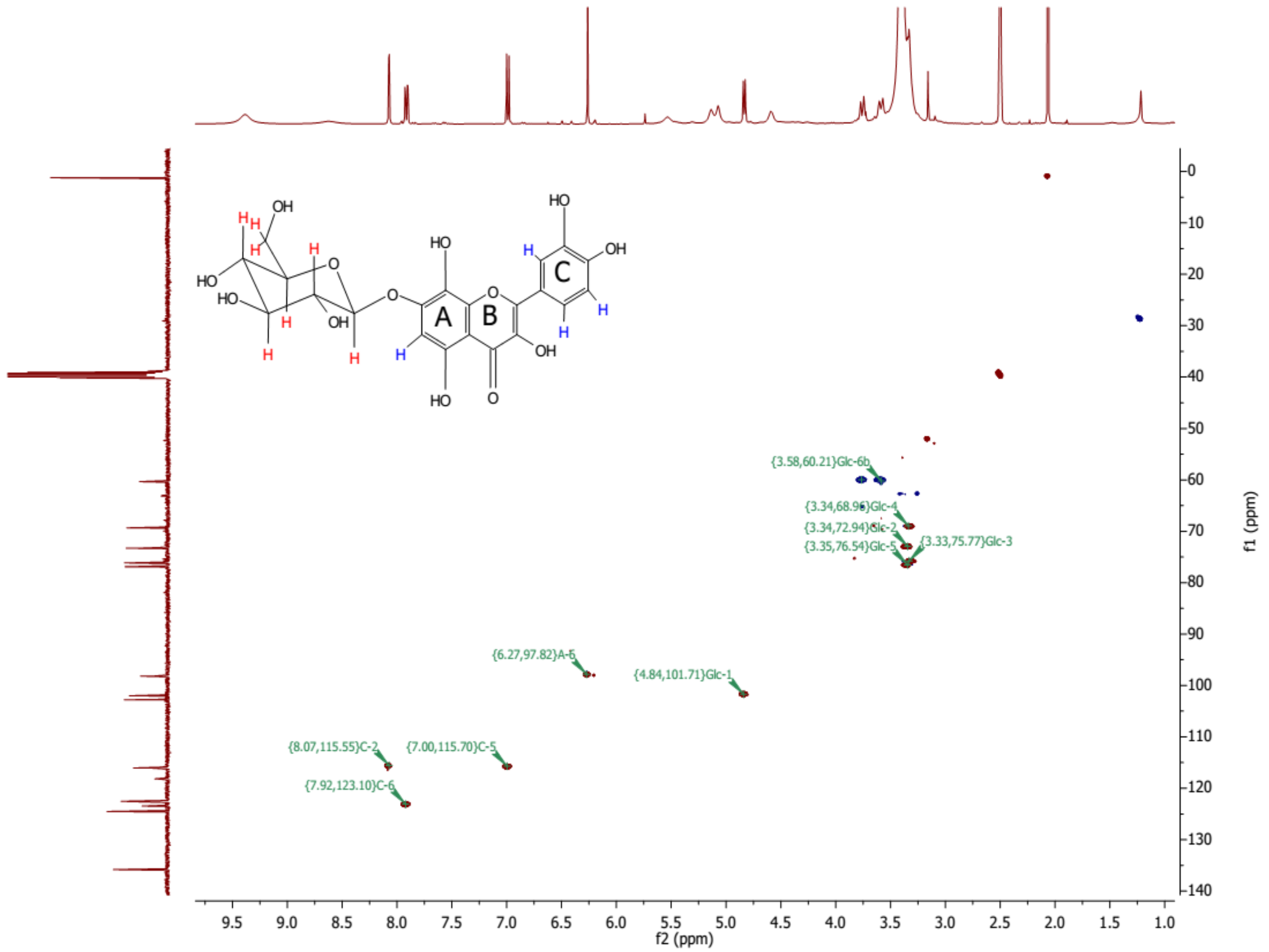


**Figure S3.** HSQC spectrum of gossypitrin in DMSO-$d_6$.

In table 1, NMR data collected verified the structure of the gossypitrin molecule in comparison with previous report (Schliemann et al., 2006).

**Table 1.** $^{1}$H (400 MHz) and $^{13}$C (100 MHz) data of gossypitrin

| No. | DMSO-$d_6$ | | Schliemann et al., 2006 | |
|---|---|---|---|---|
| | δ, mult., *J*(Hz) | δ[a] | δ (125 MHz) | |
| 2 | | 146.0 | 147.3 | |
| 3 | 9.41, s | 135.7 | 135.6 | |
| 4 | | 176.1 | 175.9 | |
| 5 | | 153.1 | 151.0 | |
| 6 | 6.26, s | 98.0 | 97.8 | |
| 7 | | 152.4 | 150.0 | |
| 8 | | 124.4 | 126.6 | |
| 9 | | 145.0 | 143.1 | |
| 10 | | 102.7 | 104.4 | |
| 1′ | | 123.3 | 122.0 | |
| 2′ | 8.08, d | 115.7 | 115.2 | |
| 3′ | | 145.1 | 144.7 | |
| 4′ | | 148.6 | 147.5 | |
| 5′ | 6.97, d | 115.9 | 115.6 | |
| 6′ | 7.89, dd | 122.4 | 120.3 | |
| 1″ | 4.84, d | 101.8 | 101.4 | |
| 2″ | 3.74, m | 73.2 | 73.2 | |
| 3″ | 3.76, m | 76.0 | 75.3 | |
| 4″ | 3.14, m | 69.2 | 69.6 | |
| 5″ | 3.32, m | 76.7 | 77.3 | |
| 6″A | | 60.6 | 60.2 | |
| 6″B | | | | |

[a] Chemical shift obtained from HSQC and HMBC spectra

Chemical shifts from the glycoside protons: δ = 5.56 ppm, δ = 5.11 ppm, δ = 4.82 ppm and δ = 4.63 ppm. Exchangeable phenolic signals of aglycone: δ = 11.89 (s, 5-OH), δ = 10.5 (s, 4′-OH), δ = 9.44 (s, 3′-OH), δ = 8.66 (s, 8-OH).

**Reference**

Schliemann, W.; Schneider, B.; Wray, V.; Schmidt, J.; Nimtz, M.; Porzel, A.; Böhm, H.,. Flavonols and an indole alkaloid skeleton bearing identical acylated glycosidic groups from yellow petals of Papaver nudicaule. *Phytochemistry* **2006**, *67*, 191–201. DOI: 10.1016/j.phytochem.2005.11.002
